# Arthur Martineau

**Published:** 1911-05-20

**Authors:** 


### Obituary.

On May 11, Arthur Martineau, M.R.C.S., L.R.C.P., assistant medical officer at Bodmin County Asylum, younger son of E. K. Martineau, of Edgbaston, aged thirty-six.

